# Haematological indices and immune response profiles in dogs naturally infected and co-infected with *Dirofilaria repens* and *Babesia canis*

**DOI:** 10.1038/s41598-023-29011-2

**Published:** 2023-02-04

**Authors:** Dagmara Wężyk, Karolina Romanczuk, Anna Rodo, Dziyana Kavalevich, Anna Bajer

**Affiliations:** 1 Department of Eco-Epidemiology of Parasitic Diseases, Institute of Developmental Biology and Biomedical Sciences, Faculty of Biology, University of Warsaw, Miecznikowa 1 Street, 02-096 Warsaw, Poland; 2 Department of Cytology, Institute of Developmental Biology and Biomedical Sciences, Faculty of Biology, University of Warsaw, Miecznikowa 1 Street, 02-096 Warsaw, Poland; 3 Department of Pathology and Veterinary Diagnostics, Warsaw University of Life Sciences-SGGW, 159C Nowoursynowska Street, 02-766 Warsaw, Poland; 4 Lab-Wet, Veterinary Diagnostic Laboratory, ul. Wita Stwosza 30 Street, 02-661 Warsaw, Poland

**Keywords:** Immunology, Molecular biology, Zoology

## Abstract

Co-infections with *Dirofilaria repens* and *Babesia canis* are rarely reported in the literature and there is very limited knowledge of their impact on canine health. Central Poland is endemic for both parasites, posing a risk of co-infections in dogs. To evaluate the impact of co-infection with *B. canis* and *D. repens* on canine health, four groups of dogs were examined: healthy dogs, dogs infected with *B. canis*, dogs infected with *D. repens* and dogs co-infected with both species. Blood parameters indicative of anaemia, kidney and liver damage were analysed statistically. Additionally, expression levels of immune response genes were quantified and compared, to define the type of immune response typically encountered in single- and co-infections. In dogs infected with *D. repens*, no major alterations in blood parameters were observed. Dogs infected with *B. canis* suffered from anaemia, kidney and liver insufficiency. In contrast, dogs co-infected with *D. repens* and *B. canis* showed milder alternation in blood biochemical parameters associated with liver (ALP activity) and kidney (serum urea and creatinine levels) dysfunction, compared to dogs infected only with *B. canis*. The expression of genes associated with cellular (Th1-mediated) (*STAT4* and *INF-γ*), humoral (Th2-mediated) (*STAT6*, *GATA3*, *SOCS3, IL-13*) and regulatory (*IL-10*) responses was quantified. For this analysis, dogs infected with *B. canis* were divided into two groups—‘Babesia 1’ (mild babesiosis), ‘Babesia 2’ (severe babesiosis). All the tested factors, except *INF-γ,* were found to be expressed in dogs infected with *D. repens*. In ‘Babesia 1’ dogs, expression of *GATA3* was highest, while in ‘Babesia 2’—*INF-γ* and *SOCS3* dominated. *IL-13* expression was predominant in dogs infected with *D. repens*, and *STAT6* and *IL-10* predominated in dogs with co-infections.

## Introduction

*Babesia canis* and *Dirofilaria repens* are widespread parasites of dogs, with expanding geographical distribution in Central and North-Eastern Europe^1–9^. Babesiosis due to *B. canis* infection is an emerging tick-borne disease^1,2,10,11^. The most common symptoms of babesiosis are lethargy, fever, anorexia, icterus and exercise intolerance^11–13^. While some infected dogs remain asymptomatic or present with only mild transient symptoms^1,11,14^, the outcome of infection can be fatal in dogs with severe babesiosis, and despite treatment, fatality ranges 2–10%^1,2^. Some dogs may develop shock and/or renal failure leading to death^12,15–18^. Following the acute phase of infection, piroplasm infections may persist in a host in chronic form^19–21^.

The diverse course and outcomes of *B. canis* infections have led to the assumption that it is not the impact of the parasite itself (causing direct cell damages and local inflammatory process) but rather the secondary immunopathology (e.g. the induction of autoantibodies or formation and deposition of immune complexes of antigen, antibody and complement) that is responsible for the major pathological consequences of infection^17,22^. The host immune response to *Babesia* spp. is not well recognized due to a range of factors influencing the response, including different phases of infection (colonization, acute phase, resolution and/or transition to chronic phase), different course/outcome of infection, possible co-infections and individual differences observed in experimental infections in dogs^22–24^. As intraerythrocytic parasites, babesia are believed to induce a protective Th1-regulated cellular immune response with interferon gamma (*IFN-γ*) playing a key role in clearance of parasites from the blood^17,22,25,26^. However, host immunity in vector-borne canine diseases is often dominated by Th2-regulated humoral responses rather than the Th1-regulated cellular arm of the immune system^22^. This allows persistence of infection and facilitates the development of the inappropriate secondary immunopathology referred to above, characterised by hypergammaglobulinemia, autoantibody and immune complex formation, contributing to further pathologies^17^. Therefore, in dogs infected with *B. canis,* we might expect different levels of expression of a wide range of cytokines and transcription factors, from the pro-inflammatory cytokine *IFN-γ*, tumour necrosis factor (*TNF-α*), interleukin (*IL-2*), the immunosuppressive *IL-10*, to cytokines associated with Th2 immune responses (i.e. *IL-4, IL-5, IL-10* and *IL-13*)^17^. Moreover, we might expect more complex expression of immune factors in dogs with a range of babesiosis symptoms than in asymptomatic infected dogs, as has been found in dogs diagnosed with leishmaniasis^22^.

In the case of *D. repens*, long-term asymptomatic infection may occur in dogs, and the reported symptoms are usually mild^4,27–29^. The vectors of *D. repens* are mosquitoes of the family *Culicidae*^30–32^. The mosquito, by feeding on the blood of an infected individual, uptakes the L1 stage larvae (microfilariae), which move into the mosquito's gut within 1–3 days. From there, they migrate to the Malpighian tubules, where they moult twice to develop into L3 stage larvae. L3 migrate to the mosquito's mouth organs and during next blood uptake are inoculated into the dermis of the definitive host where, after several months of migration, they settle under the skin (in subcutaneous or connective tissues), mature and reproduce^6,8^. Females release microfilariae, which circulate in canine blood^33,34^. The presence of adult nematodes may be associated with the formation of subcutaneous nodules, but apparently may also not cause a local inflammatory process. Some dogs remain microfilaraemic for a long period (patent infection) but some may be amicrofilaraemic (occult infections)^35^. MFs were also found in the parenchyma and/or blood vessels in numerous vital organs of dogs and their presence was associated with necrotic foci and infiltration of inflammatory cells (particularly eosinophils) in the liver, kidneys and the heart^36^. Over the course of several years of infection (2–5 years)^29,37^ *D. repens* females produce new generations of microfilariae (MF) and their concentration in the blood may achieve high levels^38^. The co-occurrence of *Babesia* and MF in circulating blood might contribute to pronounced changes in blood indices of co-infected dogs. Studies on pathology caused by *D. repens* in dogs are scarce^36,39^, as is knowledge on induced immune responses. Much more is known about immune response to more pathogenic *Dirofilaria immitis*, causing pulmonary dirofilariasis^35,40,41^.

In contrast to viruses, bacteria or protists, helminths usually cause long-lasting infections and promote Th2-regulated immune responses^42–45^. This is possible due to their immunomodulatory effects on hosts, the main strategy for their survival^43^. Filariae cause long-lasting chronic infections in humans and animals via the production of a range of immunomodulatory molecules affecting host responses^45,46^. Interestingly, alternation of immune response types has been observed in numerous previous studies of co-infections with protists and helminths, which have provided evidence for either deepened or mitigated pathology in such co-infections^46,47^. Thus, a highly relevant question is how this modulation influences the host in terms of reduced or increased immune pathology and host survival^45^.

Our aim was to determine the effect of co-infection with *D. repens* and *B. canis* on canine health. Our studies on dogs in central Poland in 2015 revealed a very high rate of co-infections with *B. canis* and *D. repens* in dogs treated for babesiosis and lack of asymptomatic co-infections in healthy dogs^48^. Contrary to our predictions, the values of biochemical parameters in dogs with co-infections were closer to those of healthy dogs than those solely infected with *B. canis*, suggesting milder babesiosis in these animals^48^. These findings are important due to the current spread of dirofilariosis in central Europe, which being mostly undiagnosed has resulted in large numbers of untreated microfilaremic dogs in the region that may be more prone to babesiosis. The possible protective effect of the nematode infection on hepatic or renal dysfunction in canine babesiosis is intriguing and its mechanisms require further investigation.

We expected *D. repens* mainly to activate Th2-regulated immune responses as in other filarial nematodes and patent *D. immitis* infections^35,42,46^. Therefore, in the current study we tested this hypothesis, predicting that infection with filariae would drive the host response towards Th2-type immunity. This in turn should result in a reduction of proinflammatory Th1-type responses that are crucial for the elimination of intracellular pathogens, including protozoa such as *B. canis*.

## Methods

### Material

A total of 140 EDTA-fixed blood samples were obtained in 2018–2020 through cooperation with veterinary practitioners and the veterinary diagnostic laboratory Lab-Wet. Samples were collected in Masovia, Central Poland, the region that is endemic for both *D. repens* and *B. canis*^4,29,48^. Criteria for inclusion of samples in the study were: (1) samples collected from clinically healthy dogs, older than 1 year; (2) samples collected from dogs with symptoms of babesiosis with laboratory confirmed *B. canis* infection.

Inclusion criteria were not based on *D. repens* infection status, thus all obtained samples were examined for *Dirofilaria* infection by molecular methods, PCR amplification and sequencing of two genetic markers, a 320 bp fragment of 12S rDNA^48^ and an approximately 600 bp-long fragment of the NADH gene^4^. To confirm *B. canis* infection in dogs with babesiosis symptoms and to detect any asymptomatic infections, all samples were also tested for *B. canis* by PCR amplification and sequencing of two markers, a 550 bp fragment of 18S rDNA^49^ and a 330 bp fragment of the mitochondrial cytochrome c oxidase subunit 1 (cox1)^50^. On this basis, dogs were allocated to four groups: control healthy dogs (n = 39), dogs infected with *B. canis* (n = 82), dogs infected with *D. repens* (n = 12) and dogs co-infected with *B. canis* and *D. repens* (n = 7). Complete results of haematological investigations (a range of haematological indices, including blood counts, biochemistry, etc.; (Table 1) were provided for these 140 dogs by two diagnostic laboratories (Lab-Wet, VetLab). A subset of 54 samples (three healthy control dogs, 32 selected dogs with babesiosis, 12 dogs infected with *D. repens* and seven dogs co-infected with *B. canis* and *D. repens*) were involved in the study on the expression levels of selected cytokines/transcription factors. Due to financial limits, it was not possible to examine expression in all *Babesia*-positive dogs, thus dogs representing mild and severe disease were chosen randomly.

**Table 1:** Comparison of blood parameters between healthy dogs, infected with one parasite, and dogs with co-infection.

Parameter	Mean parameter value ± SEM				Reference values	The value of the F statistic	P
	Healthy dogs (N = 39)	Dogs with *B. canis* (N = 82)	Dogs with *D. repens *(N = 12)	Dogs with co-infection (N = 7)			
Morphological indices							
Erythrocytes (T/l)	7.06 ± 0.210	5.05 ± 0.145^a^	6.16 ± 0.379	5.77 ± 0.496	5.50–8.50	F_3, 139_ = 22.64	< 0.001
Leukocytes (G/l)	9.56 ± 0.788	5.32 ± 0.543^a^	9.53 ± 1.420	5.32 ± 1.859^a^	6.0–12.0	F_3, 139_ = 9.23	< 0.001
Hemoglobin (mmol/l)	10.54 ± 0.229	7.49 ± 0.206^a^	8.18 ± 0.539^a^	8.61 ± 0.706^a^	9.30–11.8	F_3, 139_ = 26.22	< 0.001
Hematocrit (%)	46.55 ± 2.823	22.48 ± 1.947^a^	37.65 ± 5.089^a^	36.14 ± 6.664^a^	44.0–55.0	F_3, 139_ = 17.29	< 0.001
MCV (fl)	70.09 ± 1.050	69.86 ± 0.724	69.13 ± 1.892	71.31 ± 2.477	60.0–77.0	F_3, 139_ = 0.191	0.920
MCH (pg)	23.02 ± 1.677	16.62 ± 1.157^a^	19.53 ± 3.024	21.16 ± 3.959	19.0–24.0	F_3, 139_ = 6.19	< 0.001
MCHC (g/dl)	33.41 ± 1.231	29.95 ± 0.849	31.36 ± 2.219^a^	32.20 ± 2.905	32.0–36.0	F_3, 139_ = 6.00	0.002
Thrombocytes (g/l)	228.64 ± 17.945	27.59 ± 12.375^a^	206.75 ± 32.35	31.57 ± 42.356^a^	150.0–500.0	F_3, 139_ = 55.83	< 0.001
Biochemical indices							
Serum glucose (mg/dl)	61.48 ± 7.095	78.67 ± 5.117	51.38 ± 13.36^a^	74.66 ± 16.748	54.9–109.8	F_3, 129_ = 1.47	0.192
Creatinine (mg/dl)	1.00 ± 0.162	1.14 ± 0.115	0.92 ± 0.306	1.01 ± 0.383	0.396–1.49	F_3, 132_ = 0.26	0.935
Serum urea (mg/dl)	31.85 ± 12.010	76.61 ± 8.548^b^	40.08 ± 22.615	77.08 ± 28.349^b^	19.9–50.0	F_3, 132_ = 2.38	0.041
Total serum protein (g/l)	66.32 ± 1.202	54.81 ± 0.855	69.00 ± 2.263	60.18 ± 2.837	54.0–75.0	F_3, 98_ = 16.5	< 0.001
AST (U/l)	36.31 ± 15.233	101.87 ± 10.98^b^	41.14 ± 28.68	80.90 ± 35.955^b^	1.0–76.0	F_3, 130_ = 2.82	0.018
ALT (U/l)	37.68 ± 11.391	67.92 ± 8.160	40.06 ± 21.44	116.63 ± 26.88^b^	1.0–80.0	F_3, 131_ = 2.70	0.023
ALP (U/l)	53.39 ± 12.311	154.05 ± 8.762^b^	81.52 ± 23.18	85.09 ± 29.059	1.0–141.0	F_3, 130_ = 11.30	< 0.001

^a^Decreased blood parameters.

^b^Increased blood parameters.

### RNA extraction, reverse transcription and qPCR

400 μl of EDTA- fixed whole blood samples were centrifuged for 10 min at a temperature of 4 °C to remove red blood cells. The supernatant was harvested and then total RNA was isolated using the mirVana™ miRNA Isolation Kit (Thermo Fisher Scientific, Waltham, Massachusetts, U.S.) according to the manufacturer’s instructions. RNA was quantified using Nanodrop One (Thermo Fisher Scientific). Reverse transcription was conducted to obtain cDNA for qPCR analysis, using at least 100 μg of total RNA. cDNA was synthesized using the RevertAid First Strand cDNA Synthesis Kit (Thermo Fisher Scientific, Waltham, Massachusetts, U.S.) according to the manufacturer’s instructions. The quantitative polymerase chain reaction (qPCR) assays were performed using TaqMan Gene Expression Master Mix (Thermo Fisher Scientific, Waltham, Massachusetts, U.S.) and the following TaqMan probes: Cf02623316_m1 (*IFN-γ*), Cf02624080_m1 (*IL-13*), Cf02624265_m1 (*IL-10*), Cf02741636_m1 (*GATA3*), Cf02676700_m1 (*STAT4*), Cf01127458_m1 (*STAT6*), Cf02690456_g1 (hypoxanthine–guanine phosphoribosyl transferase, HPRT). HPRT expression was used as an endogenous reference gene. Data were normalized to the expression observed in a group of three healthy dogs, whose blood parameters were within the normal range. Gene expression analysis was performed in duplicates. All qPCR reactions were performed on a LightCycler96 instrument (Roche, Basel, Switzerland). The amplification curves were analysed using LightCycler 96 software (Roche) to determine the Ct values. According to the Livak and Schmittgen method, the relative quantification was calculated using 2−ΔΔCt analysis^51^. Results are presented as means and standard errors of the mean (SEM).

### Statistical analysis

Statistical analyses for morphological and biochemical blood indices were performed using IBM SPSS v. 26 software. General Linear Models (GLM) were implemented with morphological and biochemical blood parameters as dependent variables and infection status in dogs as the explanatory factor (four levels corresponding to four groups of dogs: healthy dogs, dogs with *B. canis* infection, dogs with *D. repens* infections, dogs with co-infection of *B. canis* and *D. repens*).

In a further stage of analysis, severity of symptoms of babesiosis (the magnitude of changes in haematological and biochemical indices) was included as an explanatory factor in GLMs. For this analysis the dogs were allocated to three classes of severity of babesiosis (described below) and statistical analysis (GLM) was performed to confirm correct categorization of scores (Table 2). The presence/absence of *D. repens* infection, was fitted as a binary factor (infected = 1, not infected = 0) in further GLMs, incorporating also severity scores of babesiosis (1–3) as dependent variables (anaemia, hepatic or renal dysfunction, see below). Classification of severity was based on the criteria provided in Bajer et al. (2016)^48^. The three main groups of general symptoms (indicators of mortality in canine babesiosis) included: a. severity of anaemia (severity score 0—RBC counts within normal range 5.5–8.0 T/l; severity score 1—RBC counts in a range 4.5–5.5 T/l; severity score 2—RBC below 4.5 T/l); b. hepatic dysfunction parameters (measures of enzymes activity) based on reference values for AST– aspartate aminotransferase, ALT- alanine aminotransferase and ALP- alkaline phosphatase within, above (up to 100 U/l) and profoundly above (> 100 U/l) the normal range, representing scores of 0, 1 and 2, respectively (Table 2); c. renal dysfunction parameters (based on reference values for serum urea) within, above (up to 100 mg/dl) and profoundly above (> 100 mg/dl) the normal range, representing severity scores of 0, 1 and 2, respectively (Table 2).

**Table 2:** Comparison of selected blood parameters in classifying the severity of the main symptoms in canine babesiosis.

Symptoms and associated blood parameters ± SEM	Severity class			Reference values	Value of statistics F	P
	Class 0 (normal)	Class 1 (mild)	Class 2 (advanced disorder)			
Anemia	N = 101	N = 38	N = 31			
Erythrocytes (T/l)	6.75 ± 0.79	5.06 ± 0.129	3.39 ± 0.142	5.50–8.50	F_2, 169_ = 231.75	< 0.001
Leukocytes (G/l)	8.09 ± 0.562	7.17 ± 0.917	8.12 ± 1.015	6.0–12.0	F_2, 169_ = 0.40	0.673
Hemoglobin (mmol/l)	9.96 ± -0.122	7.56 ± 0.199	5.21 ± 0.220	9.30–11.8	F_2, 169_ = 194.04	< 0.001
Hematocrit (%)	39.51 ± 1.778	26.48 ± 2.899	15.90 ± 3.210	44.0–55.0	F_2, 169_ = 23.28	< 0.001
Thrombocytes (g/l)	164.40 ± 14.680	151.23 ± 23.932	28.10 ± 26.497	150.0–500.0	F_2, 169_ = 10.36	< 0.001

Hepatic dysfunction	N = 80	N = 48	N = 33			
AST (U/l)	42.50 ± 10.146	82.53 ± 13.099	140.56 ± 15.798	1.0–76.0	F_2, 160_ = 13.90	< 0.001
ALT (U/l)	38.10 ± 7.330	65.49 ± 9.366	124.46 ± 11.413	1.0–80.0	F_2, 161_ = 20.28	< 0.001
ALP (U/l)	68.89 ± 6.229	124.67 ± 8.049	249.81 ± 9.960	1.0–141.0	F_2, 160_ = 117.93	< 0.001

Renal dysfunction	N = 106	N = 33	N = 24			
Creatinine (mg/dl)	0.899 ± 0.093	1.003 ± 0.167	1.795 ± 0.195	0.396–1.49	F_2, 162_ = 8.62	< 0.001
Serum urea (mg/dl)	31.69 ± 5.180	70.06 ± 9.284	190.40 ± 10.887	19.9–50.0	F_2, 162_ = 87.02	< 0.001

For the next step of analysis, 82 dogs infected with *B. canis* were divided into three classes of severity of babesiosis (Table 3). All dogs with any of these three general pathologies classified as ‘severity score 2’ were assigned to group 3 (severe babesiosis, severity level 3, n = 44). Dogs presenting with more than one dysfunction (anaemia, signs of hepatic or renal failure) at level 1 (abnormal) were assigned to group 2 (moderate babesiosis, severity level 2, n = 16) and dogs presenting with one group of signs (mainly mild anaemia and thrombocytopenia) were assigned to group 1 (mild babesiosis, severity level 1, n = 22).

**Table 3:** Comparison of blood parameters between healthy dogs, infected with one parasite, and dogs with co-infection in different classes of symptom severity.

Level	Mean blood parameters ± SEM	Reference values	Healthy dogs (N = 39)	Dogs with *B. canis *(N = 82)	Dogs with *D. repens *(N = 12)	Dogs with coinfection (N = 7)	Value of statistics F	P
Anemia								
Level 0	Erythrocytes (T/l)	5.50–8.50	7.06 ± 0.12	6.28 ± 0.13	6.91 ± 0.26	6.59 ± 0.378	F_10, 137_ = 7.08 F_10, 137_ = 2.06 F_10, 137_ = 0.94 F_10, 137_ = 3.44 F_10, 137_ = 0.75	0.611 0.003 0.47 0.06 < 0.001
	Leukocytes (G/l)	6.0–12.0	9.56 ± 0.74	5.24 ± 0.80	10.89 ± 1.65	2.86 ± 2.337		
	Hemoglobin (mmol/l)	9.30–11.8	10.54 ± 0.18	9.14 ± 0.19	9.88 ± 0.40	9.48 ± 0.571		
	Hematocrit (%)	44.0–55.0	46.55 ± 2.63	25.59 ± 2.82	47.53 ± 5.82	44.06 ± 8.234		
	Thrombocytes (g/l)	150.0–500.0	228.64 ± 15.7	38.36 ± 16.87	219.62 ± 34.7	37.32 ± 49.18		
Level 1	Erythrocytes (T/l)		–	4.99 ± 0.15	5.15 ± 0.53	4.65 ± 0.53		
	Leukocytes (G/l)		–	4.38 ± 0.95	8.96 ± 3.30	4.30 ± 3.30		
	Hemoglobin (mmol/l)		–	7.49 ± 0.23	7.44 ± 0.80	6.60 ± 0.80		
	Hematocrit (%)		–	24.36 ± 3.36	35.55 ± 11.64	0.33 ± 0.67		
	Thrombocytes (g/l)		–	16.77 ± 20.08	329.5 ± 69.56	39.0 ± 59.56		
Level 2	Erythrocytes (T/l)		**–**	3.36 ± 0.15	4.14 ± 0.53	4.01 ± 0.75		
	Leukocytes (G/l)		**–**	6.38 ± 0.95	4.65 ± 3.30	4.66 ± 4.67		
	Hemoglobin (mmol/l)		**–**	5.16 ± 0.23	5.95 ± 0.80	5.98 ± 1.14		
	Hematocrit (%)		**–**	16.21 ± 3.36	29.0 ± 11.6	9.96 ± 11.44		
	Thrombocytes (g/l)		**–**	23.12 ± 20.08	32.50 ± 69.56	23.01 ± 28.37		
Hepatic dysfunction								
Level 0	AST (U/l)	1.0–76.0	36.32 ± 5.76	52.16 ± 7.50	41.87 ± 12.00	51.45 ± 25.45	F_10, 127_ = 2.94 F_10, 129_ = 4.77 F_10, 128_ = 0.99	0.01 < 0.001 0.429
	ALT (U/l)	1.0–80.0	37.68 ± 9.77	37.81 ± 12.73	37.80 ± 20.35	43.30 ± 43.18		
	AP (U/l)	1.0–141.0	53.39 ± 8.29	98.58 ± 10.83	57.00 ± 17.27	65.00 ± 36.63		
Level 1	AST (U/l)		–	86.99 ± 6.26	43.90 ± 36.00	69.1 ± 18.00		
	ALT (U/l)		–	58.10 ± 10.47	61.60 ± 61.06	67.95 ± 30.53		
	AP (U/l)		–	129.92 ± 8.88	175.30 ± 51.8	86.15 ± 25.90		
Level 2	AST (U/l)		**–**	134.08 ± 8.48	31.70 ± 36.00	187.0 ± 36.00		
	ALT (U/l)		**–**	121.94 ± 14.0	38.90 ± 61.06	458.00 ± 61.06		
	AP (U/l)		**–**	258.82 ± 11.5	208.40 ± 51.8	121.00 ± 51.80		
Renal dysfunction								
Level 0	Creatinine (mg/dl)	0.396–1.49	1.00 ± 0.16	0.88 ± 0.156	0.88 ± 0.35	1.03 ± 0.49	F_10, 130_ = 0.13 F_10, 130_ = 0.81	0.992 0.562
	Serum urea (mg/dl)	19.9–50.0	30.84 ± 8.84	34.10 ± 8.619	28.29 ± 19.2	36.85 ± 27.25		
Level 1	Creatinine (mg/dl)		0.97 ± 0.98	1.04 ± 0.22	1.00 ± 0.57	1.20 ± 0.98		
	Serum urea (mg/dl)		69.95 ± 54.51	73.05 ± 12.18	71.53 ± 31.47	74.77 ± 54.51		
Level 2	Creatinine (mg/dl)		**–**	1.87 ± 0.24	**–**	1.24 ± 0.69		
	Serum urea (mg/dl)		**–**	180.80 ± 13.2	**–**	158.71 ± 38.55		

Statistical analyses for comparison of gene expression levels were performed with GraphPad Prism 9.4.0. Shapiro–Wilk and Kruskal–Wallis tests were used to compare relative mean expression levels between the groups. The level of significance was set at *P* ≤ 0.05.

### Ethics approval and consent to participate

The study was carried out on blood samples provided voluntarily by dog owners, thus no ethical approval/ license was required for this study (as per Resolution on the protection of animals used for scientific or educational purposes, 15th January 2015 [Dz. U. 2015 position 266] Chapter 1, Paragraph 1.2.1). The owners of dogs involved in this study were informed about the aims of the study, provided oral consent and contact information to obtain the results of testing. Haematological tests for these dogs were conducted as commercial service by VetLab diagnostics laboratory (Warsaw, Poland) and costs of testing were covered by the project. Additionally, blood remnants obtained after testing were provided by dr Anna Rodo from Lab-Wet diagnostic laboratory (Warsaw, Poland), co-author of the current paper, with the consent from the head of Lab-Wet unit in Warsaw.

## Results

### Prevalence of *Dirofilaria repens*

The DNA of *D. repens* was detected in 19 of 140 examined dogs giving an overall prevalence of 13.5% (combined dogs with single- and co-infections). Prevalence was 23.5% among 51 ‘healthy’ dogs and 7.8% among 89 dogs infected with *B. canis*.

### Effect of parasite infection on haematological parameters

A range of haematological parameters was compared between four groups of dogs. The mean values of blood counts and biochemical parameters with relevant statistics are presented in Table 1.

The highest number of haematological abnormalities was observed in the group of dogs infected with *B. canis*: decreased mean number of erythrocytes, leukocytes, platelets, haemoglobin concentration, haematocrit, MCH, MCHC, and increased concentrations of serum urea and activity of the liver enzymes, AST and ALP (Table 1). Moreover, in this group of dogs mean values for erythrocytes, platelets, haemoglobin, haematocrit, MCH and MCHC were the lowest, and mean values for liver enzyme activity for ALP and AST the highest.

The lowest number of haematological alterations in comparison to the other groups was observed in the group of dogs infected only with *D. repens*: reduced mean concentrations of haemoglobin and glucose, decreased haematocrit and MCHC (Table 1). In dogs co-infected with *B. canis* and *D. repens*, alterations in seven blood indices were recorded: reduced mean numbers of leukocytes, platelets, concentration of haemoglobin, haematocrit value, and an increased concentration of serum urea and elevated activity of the liver enzymes AST and ALT (Table 1).

A classification of the severity of babesiosis symptoms for individual dogs was carried out to enable further analysis. The symptoms were compiled to create three general classes of symptoms (anaemia, symptoms of hepatic or renal failure), each with three severity levels (Table 2).

Statistical analysis of blood parameters confirmed the validity of classification based on all parameters except leukocyte counts (Table 2). A significant decrease in blood morphological parameters was observed in successive classes of severity of babesiosis based on severity of anaemia. In the case of renal and hepatic dysfunction, a more profound alteration of blood biochemical parameters was observed in successive classes of severity of babesiosis (Table 2).

In the next step, statistical comparisons of the haematological parameters were performed based on the classification of severity of different babesiosis symptoms (Table 3). Among the dogs with the most advanced anaemia (class 2), the greatest decrease in the mean RBC count and haemoglobin concentration was seen in dogs infected with *B. canis*, while the mean values of these indices were similar in dogs infected with *D. repens* and co-infected with *B. canis* and *D. repens* (Table 3). Among these groups of dogs with anaemia class 2, the lowest haematocrit and platelet count values were observed in dogs co-infected with *B. canis* and *D. repens* (Table 3).

In dogs with the most advanced alternations in enzyme activities (class 2), the pattern was diverse (Table 3). Dogs infected with *B. canis* presented with increased levels of activity of all three enzymes compared to normal values, and with the highest mean for ALP activity compared to other groups of infected dogs. Dogs co-infected with *B. canis* and *D. repens* presented with the highest mean ALT activity and elevated AST activity, and finally dogs infected with *D. repens* presented with only elevated ALP activity (Table 3).

No *D. repens*-infected dogs could be allocated to class 2 of renal disfunction based on serum urea concentration (Table 3). Among dogs with the most advanced renal dysfunction (class 2), *B. canis*-infected dogs presented with numerically higher mean serum creatinine and urea concentrations than dogs co-infected with *B. canis* and *D. repens*, although these differences were not statistically significant (Table 3).

### Immune response in dogs infected with *B. canis*, *D. repens* and co-infected with *B. canis* and *D. repens*

To identify the immune response profile in dogs infected with one parasite or co-infected with both parasites, the expression level of genes of selected cytokines and transcription factors associated with Th1 or Th2-mediated response were determined as described previously. For this analysis, *B. canis*-infected dogs with mild and moderate babesiosis (representing classes 0 and 1) were combined into the group referred to as ‘Babesia 1’ and dogs with severe babesiosis (class 2) were assigned to ‘Babesia 2’.

### Expression of cytokine genes and transcription factors related to cellular response (Th1-mediated)

**Figure 1:**
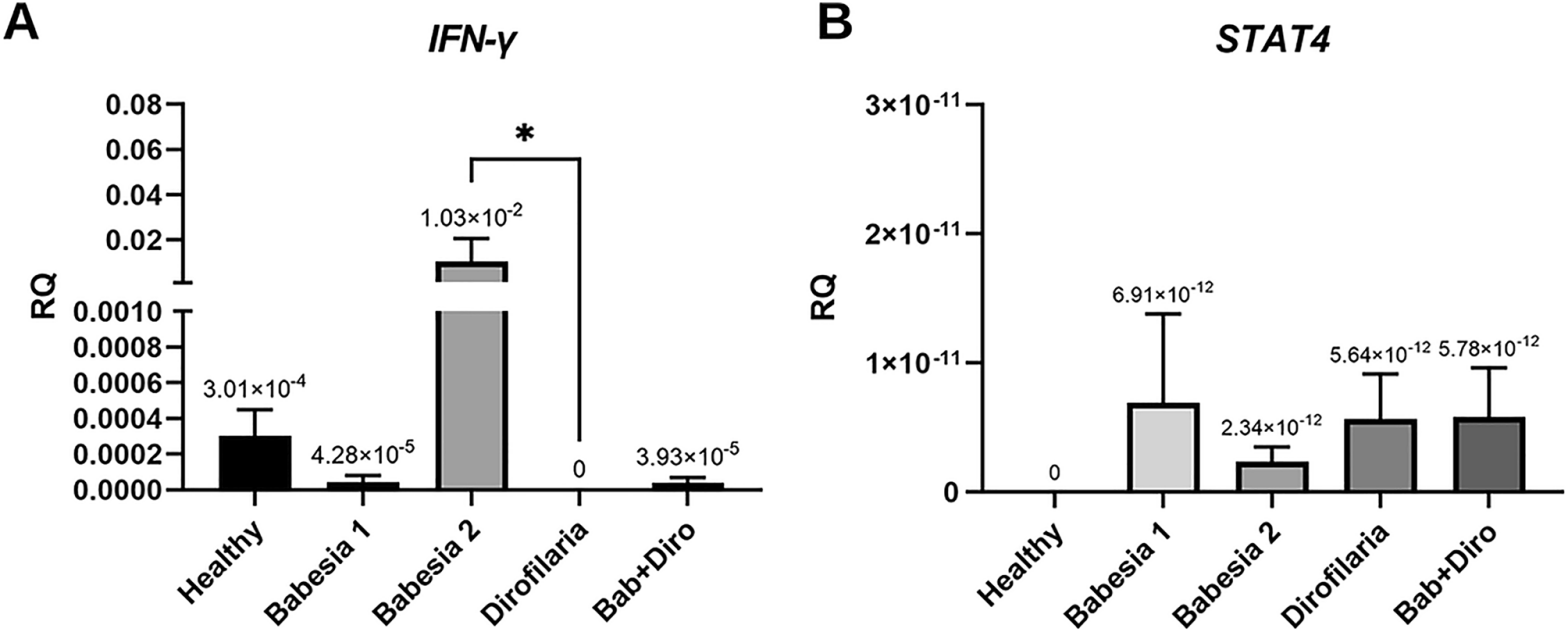
Relative expression of genes associated with Th1 response. Expression of (**A**) *INF-γ* and (**B**) signal transducer and activator of transcription *STAT4* in healthy dogs, dogs infected with *B. canis, D. repens* and co-infected with *B. canis* and *D. repens* (Bab + Diro).

*INF-γ* expression was detected in four groups (healthy dogs, dogs infected with *B. canis* [groups 1 and 2] and co-infected with *B. canis* and *D. repens*) (Fig. 1A). The expression of *INF-γ* was not detected in the group of dogs infected only with *D. repens* and expression was very low in dogs co-infected with *B. canis* and *D. repens*. The highest *INF-γ* expression was found in the group of dogs with severe babesiosis (Babesia 2) in contrast to the low expression level in dogs with mild/moderate babesiosis (Babesia 1). Significant differences in *INF-γ* gene expression were found between dogs infected with *D. repens* and dogs with severe babesiosis (Fig. 1A).

*STAT4* expression was found in four groups of dogs [dogs infected with *B. canis* (groups 1 and 2), dogs infected with *D. repens* and dogs co-infected with *B. canis* and *D. repens*] (Fig. 1B). *STAT4* gene expression was not detected in healthy dogs. Among infected dogs, the lowest level of expression was found in dogs with severe babesiosis (Babesia 2), which presented with the highest *INF-γ* expression (Fig. 1A). However, the expression of *STAT4* gene was generally similar in dogs with mild/moderate babesiosis (Babesia 1), infected with *D. repens* and co-infected with *B. canis* and *D. repens* (Bab + Diro) and differences in expression between groups were not statistically significant (Fig. 1B).

### Expression of genes of cytokines and transcription factors associated with humoral response (Th2-mediated)

**Figure 2:**
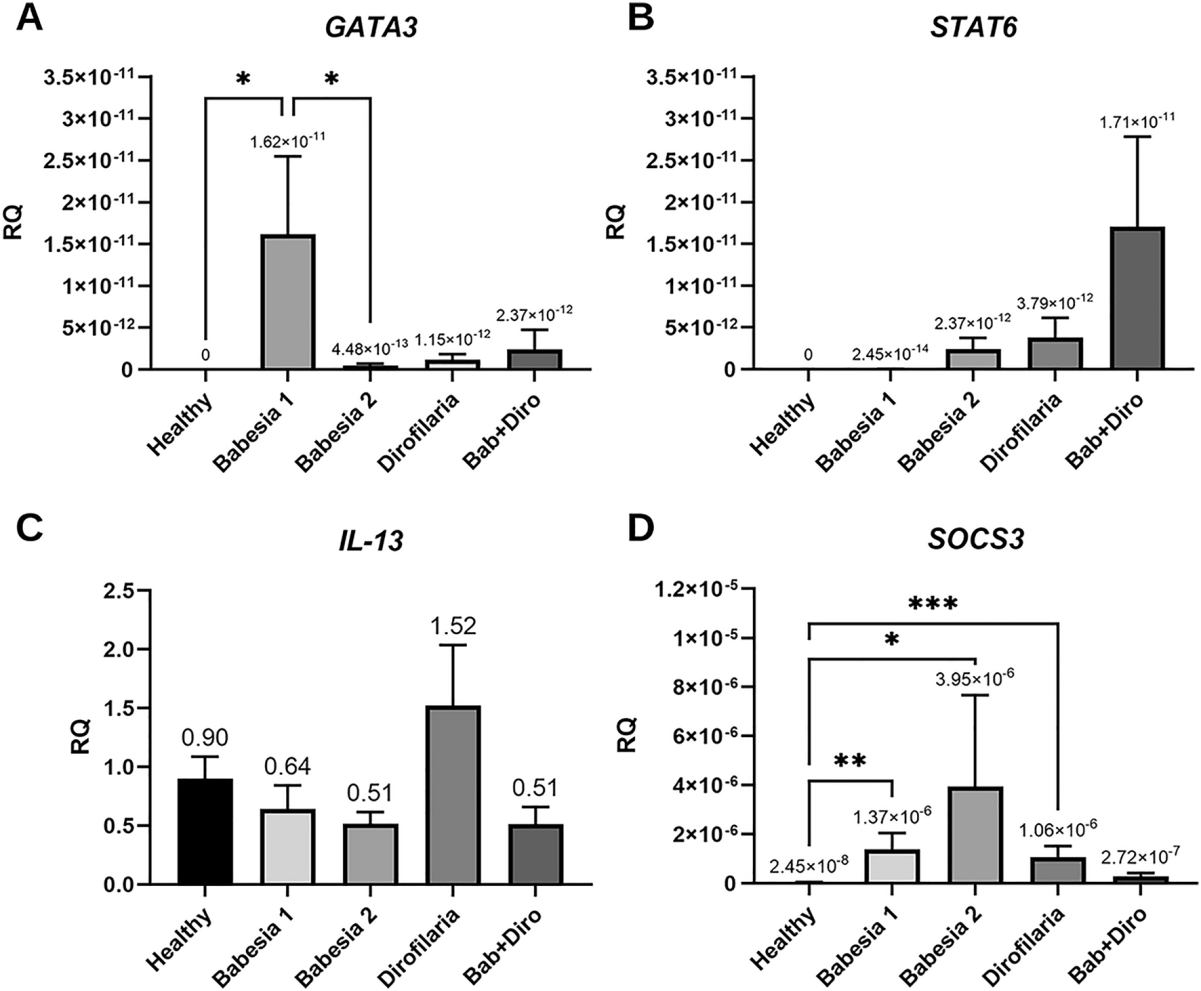
Relative expression of genes associated with Th2 and regulatory response. Expression of (**A**) transcription factor *GATA3* (**B**) signal transducer and activator of transcription *STAT6* (**C**) interleukin 13 (*IL-13*) and (**D**) suppressor of cytokine signalling (*SOCS3*) in healthy dogs, dogs infected with *B. canis, D. repens* and co-infected with *B. canis* and *D. repens* (Bab + Diro).

*GATA3* gene expression was detected in four groups of dogs [dogs infected with *B. canis* (groups 1 and 2), dogs infected with *D. repens* and dogs co-infected with *B. canis* and *D. repens*] (Fig. 2A). No expression was detected in healthy dogs. The highest level of expression was found in dogs with mild/moderate babesiosis (Babesia 1) and the lowest in dogs with advanced babesiosis (Babesia 2). Low and similar level of *GATA3* expression was found in dogs infected with *D. repens* and co-infected with *B. canis* and *D. repens* [not significant differences (NS); Fig. 2A].

The expression of the *STAT6* gene was observed in four groups of dogs [dogs infected with *B. canis* (groups 1 and 2), dogs infected with *D. repens* and dogs co-infected with *B. canis* and *D. repens*] (Fig. 2B). In healthy dogs, no *STAT6* expression was detected. The highest level of expression was found in dogs co-infected with *B. canis* and *D. repens* and the lowest in dogs with mild/moderate babesiosis (Babesia 1). Low and similar level of *STAT6* expression was found in dogs with advanced babesiosis (Babesia 2) and in dogs infected with *D. repens*. However, the differences in expression between groups were not statistically significant (Fig. 2B).

The expression of the *IL-13* gene was detected in all groups, including healthy dogs (Fig. 2C). Although the differences in *IL-13* gene expression between groups were not statistically significant, the highest level of expression (1.5 × higher than in healthy dogs), was found in dogs infected with *D. repens* (Fig. 2C).

Significant differences in the level of suppressor of cytokine signalling 3 gene (*SOCS3*) gene expression were found between the groups of dogs (Fig. 2D). The lowest level of expression was identified in healthy dogs and dogs co-infected with *B. canis* and *D. repens*. The highest level of *SOCS3* expression was detected in dogs with severe babesiosis (Babesia 2), and was relatively high also in dogs with mild/moderate babesiosis and dogs infected only with *D. repens* (Fig. 2D).

### Expression of the regulatory cytokine *IL-10* gene

**Figure 3:**
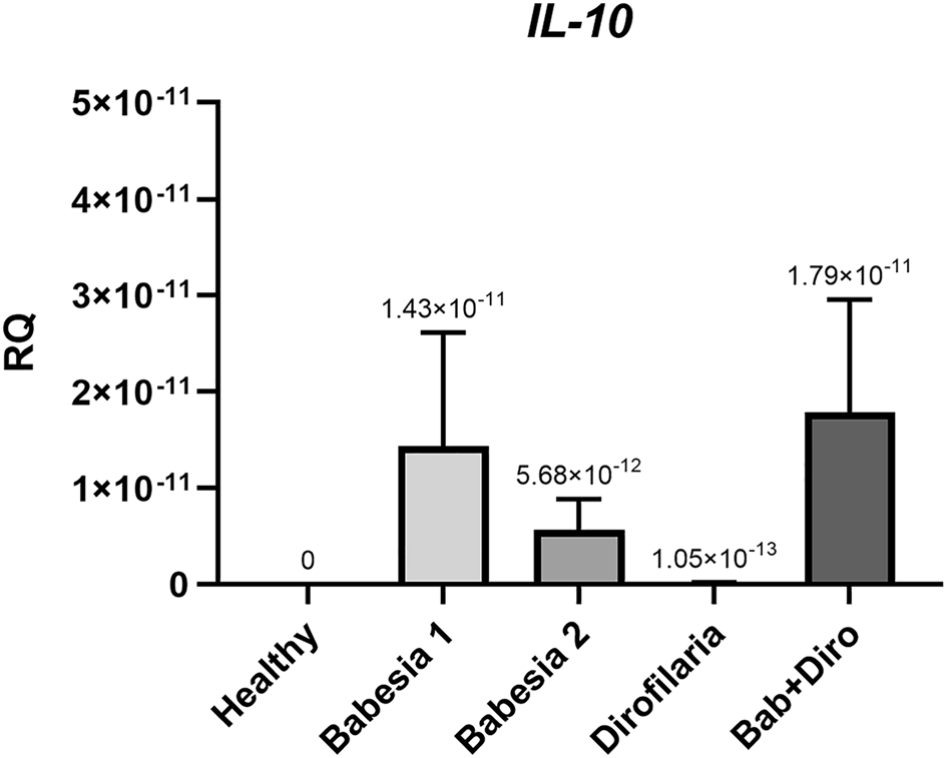
Relative expression of interleukin 10 (*IL-10*) in healthy dogs infected with *B. canis*, *D. repens*, and co-infected with *B. canis* and *D. repens* (Bab + Diro).

*IL-10* gene expression was detected in four groups of dogs [dogs infected with *B. canis* (groups 1 and 2), dogs infected with *D. repens* and dogs co-infected with *B. canis* and *D. repens*] (Fig. 3). No *IL-10* expression was detected in healthy dogs and expression level was very low in dogs with *D. repens*. The highest expression was found in dogs with co-infection. Relatively high and similar expression of *IL-10* was found in dogs with mild/moderate and severe babesiosis (Fig. 3). Differences in the level of *IL-10* gene expression between groups were not statistically significant (Fig. 3).

### Summary of immune response in dogs infected with *B. canis, D. repens* and co-infected with *B. canis* and* D. repens*

The expression of genes of all tested cytokines and related transcription factors (*STAT4, INF-γ, GATA3, STAT6, IL-10, IL-13, SOCS3*) was detected in three groups of dogs—in dogs with mild/moderate and severe babesiosis (groups Babesia 1 and Babesia 2) and in dogs co-infected with *B. canis* and *D. repens* (Figs. 1, 2, 3). The expression of all tested cytokines/factors except for *INF-γ* was observed in dogs infected only with *D. repens*.

Interestingly, there were profound differences in expression levels of cytokines/factors between dogs with mild/moderate and advanced babesiosis: the highest expression of *STAT4*, *GATA3* and *IL-10* was detected in group Babesia 1, while *INF-γ, STAT6* and *SOCS3* were expressed at a higher level in group Babesia 2.

Dogs infected with *D. repens* were characterized by slightly higher expression of *IL-13* than dogs from other groups (Fig. 2C) and also relatively high expression of *SOCS3* (Fig. 2D). Dogs co-infected with *B. canis* and *D. repens* were characterized by relatively high expression of *STAT6* and *IL-10* gene (Figs. 2B, 3).

## Discussion

The main finding of our study is the clear demonstration of significant differences in blood indices and in the expression of immune factors in dogs with single and double infections. While only minor alterations in blood parameters were observed in dogs infected with *D. repens*, dogs with *B. canis* showed more severe pathology. However, contrary to our expectations, but in agreement with our previous study^48^, dogs with co-infections of *D. repens* and *B. canis* showed fewer abnormalities in blood counts and biochemical parameters associated with liver and kidney dysfunction compared to dogs infected only with *B. canis*. Analysis of expression levels of selected transcription factors/cytokines in these co-infected dogs provided evidence of activation of Th2-mediated and regulatory (*IL-10*) immune response.

While co-infected dogs showed fewer abnormalities in blood counts and biochemical parameters compared to dogs infected only with *B. canis*, the pathology they experienced was certainly more intense than that of dogs infected only with *D. repens*. In our previous study, co-infected dogs presented with more advanced anaemia (lowest mean RBC count, lowest haemoglobin concentration and lowest haematocrit) and thrombocytopenia than dogs infected only with *B. canis*, however they presented with lower alternations in biochemical parameters^48^. In the current study, the picture was slightly different because indices of anaemia were worse in dogs infected only with *B. canis*. Mean biochemical parameters were similar in co-infected dogs and dogs only with *B. canis*, with the exception of ALP activity (twice as high in dogs with *B. canis*) and ALT activity (more elevated in dogs with co-infection). Anaemia observed in dogs with babesiosis is considered to be one of the factors causing hypoxia and hypoxic liver injury, resulting in increases in ALT, AST and ALP activities^52,53^. Both ALT and AST are present in high concentrations in hepatocytes. Any damage to hepatocytes or their membranes can result in their leakage into the circulation^54,55^. Increase in the level of ALP may be due also to damage or abnormal function of the biliary system^56^.

In analyses based on allocation of dogs to severity classes, dogs co-infected with *B. canis* and *D. repens* presented with lower indices of renal dysfunction than dogs infected only with *B. canis*. In the class with the most advanced hepatic dysfunction, dogs infected with *B. canis* presented with much higher activity of ALP than dogs co-infected by *B. canis* and *D. repens* which is in agreement with previous observations^52,57^. However, co-infected dogs presented with the highest activity of ALT and AST, reflecting possible damages to hepatocytes^52,54,55^. Thus, in the present study co-infected dogs appear to have been more resistant to development of renal failure than dogs infected only with *B. canis*^58^, although they still showed some features of hepatic injury^57^.

As expected, the highest numbers of abnormalities/alternations in blood parameters were found in dogs with babesiosis. Similar alterations in blood parameters have been observed in numerous previous studies on babesiosis due to *B. canis*^12,13,17,52,58–60^. The most common blood abnormalities occurring in the course of canine babesiosis, and also found in the present study, are thrombocytopenia, anaemia, leukopenia (although leucocytosis has also been reported previously)^13,17,52,59^.

In our previous study we had only two dogs infected solely with *D. repens*, and these presented with all blood indices within the normal range^48^. However, in the present study we analysed the blood parameters of 12 dogs infected only with *D. repens* and this time we found some alterations (decreased haemoglobin and glucose concentration, decreased haematocrit and MCHC), although these were limited in number. Interestingly, in another recent study comparing blood parameters of 197 *D. repens*-infected with those of 218 uninfected dogs from Poland^39^, no major alterations were found in infected dogs. However, as in our study, several modest but nevertheless significant differences were detected: lower mean RBC, lymphocyte and thrombocyte counts, decreased haematocrit and increased activity of ALP and creatinine were found in *D. repens*-infected dogs compared to uninfected dogs. In their further analysis of a subset of 214 dogs with haematological and biochemical results within normal reference ranges (93 *D. repens*- infected and 121 uninfected), lower numbers/values of lymphocytes, RBC and haematocrit, higher glucose concentration and borderline elevated ALP activity were observed in infected dogs compared to uninfected animals^39^. The authors concluded that despite *D. repens* infections being categorized as asymptomatic, some haematological and biochemical changes are associated with *D. repens* infections in dogs^39^. Haemoglobin levels and haematocrits below reference values were also found in our study (accompanied by decreased MCHC), and we also found slightly higher activity of all three liver enzymes in comparison to healthy dogs, but glucose concentration was decreased among our 12 *D. repens*-infected dogs. Thus, our study confirms also that unnoticed/unrecognized *D. repens* infection may have an impact on dog health.

Following the comparison of blood parameters, we identified and compared for the first time profiles of immune responses in dogs with single and double infections. Based on the literature^61^ and availability of TaqMan probes for dogs, we assessed the expression of selected Th1-related transcription factors/cytokines (*IFN-γ, STAT4*), Th2-related ones (*IL-13, STAT6, SOCS3, GATA-3*) and the T-regulatory *IL-10* in four groups of dogs. In summary, we observed the expression of all tested transcription factors/cytokines, except *INF-γ*, in dogs infected only with *D. repens*. In dogs with mild/moderate babesiosis the expression of *GATA3* was highest, while in dogs with advanced babesiosis the highest expression of *INF-γ* and *SOCS3* was recorded. Expression of regulatory *IL-10* was similar in both groups of *Babesia*-infected dogs. The expression of *IL-13* was predominant in dogs infected with *D. repens*, and the expression of *STAT6* and regulatory *IL-10* predominated in dogs with co-infection. It appears then that a somewhat mixed expression of Th1-, T-regulatory and Th2-related transcription factors and cytokines was observed both in dogs infected with *B. canis* and co-infected with *B. canis* and *D. repens*. Although we expected a mixed Th1 and Th2 immune response in co-infected dogs, the mixed immune response in *B. canis*-infected dogs was not expected. Activation of *STAT4*, involved in controlling Th1 gene expression, induces production of *IFN-γ*^62^ and as we had predicted the highest expression of *IFN-γ* was indeed observed in dogs with advanced babesiosis (Babesia 2). The higher expression of *STAT4* and *IFN-γ* is consistent with the hypothesis that in *Babesia* infections the immune response is skewed towards a Th1-profile. However, an important inhibitor of *IL-12* mediated *STAT4* activation is *SOCS3*^63^ which was found to be expressed significantly higher in dogs with advanced babesiosis (Babesia 2 group). Although *SOCS3* is expressed in both Th1 and Th2 cells, much higher expression has been reported in Th2 cells, and *SOCS3* is known to inhibit *IL-12*-mediated activation of the *STAT4* pathway, thereby inducing Th2 differentiation^63,64^. Also, *STAT6*, *GATA3* and *IL-10* expression was observed in *B. canis*-infected dogs, suggesting a switch of the immune response profile from Th1 to Th2^22,61^. *STAT6* inhibits the expression of *IFN-γ*, *IL-12* and *TNF-β* and collaborates with *GATA3*, which promotes the expression of Th2 cytokines and suppresses the expression of *IL-12* and *STAT4* (inhibiting Th1 development)^65,66^. *IL-10* is a cytokine with multiple effects on immunoregulation and inflammation and downregulates the expression of Th1 cytokines. Thus, the results of the current study do not indicate a clearly Th1-polarized profile in the course of canine babesiosis but rather a mixed expression of Th1-type and Th2-type transcription factors and cytokines. Interestingly, such a mixed profile of immune response (highest expression of either *IFN-γ* and *SOCS3*) was mostly observed in dogs with advanced babesiosis (Babesia 2), presenting with the most advanced alterations in blood counts and biochemistry, supporting the hypothesis of immune-mediated secondary pathology^22^. Furthermore, the expression of Th1 and Th2 cytokines/transcription factors in dogs with babesiosis was also accompanied by a relatively high and similar expression of regulatory *IL-10*. Interestingly, both uncomplicated and complicated babesiosis caused by *Babesia rossi* infection have been shown to induce pro‐inflammatory cytokine storms that correlate with disease severity and fatal outcomes. The authors investigated serum concentrations of 11 cytokines and generally, the more complicated the disease, the more pro‐inflammatory a cytokine profile was detected^67^. Moreover, the cytokine storm was associated with multiple organ damage during the course of *B. rossi* infection^67–69^. Thus, features of mixed Th1/Th2 -related responses in dogs infected with *B. canis* may be associated with better clinical outcomes compared to the consequences of the Th1 proinflammatory activity in dogs with babesiosis attributed to *B. rossi*.

As in *B. canis*-infected dogs, the expression of all the tested cytokines/ transcription factors was detected in dogs co-infected with *B. canis* and *D. repens.* Again, a rather mixed expression of Th1-, T-regulatory and Th2-related cytokines/transcription factors was observed in these co-infected dogs. Both *STAT6* and regulatory *IL-10* displayed highest expression which may indicate a Th2/regulatory response and may correspond to lower levels of abnormalities in blood parameters in this group of dogs. In Beagle puppies experimentally infected with the less pathogenic *Babesia gibsoni*, *IL-10* was detected only during the peak of parasitaemia and was followed by resolution of pyrexia and parasitaemia^23^. In co-infected dogs, the mixed profile of the immune response may be explained by a contribution of a filarial-induced Th2 response^43,45^. In contrast, in an experimental study on mice co-infected with filariae *Litomosoides sigmodontis* and *Leishmania major*, immune responses to *L. major* and *L. sigmodontis* were found to be highly compartmentalized and appropriately polarized, with a Th1 response to *L. major* in the popliteal lymphatic nodes (LNs) and a type 2 response to *L. sigmodontis* in the thoracic LNs^45^. Nevertheless, despite these contrasting immune responses in co-infected mice, the impact of filarial infection was still sufficient to delay footpad lesion progression. In an experimental study with *Plasmodium berghei* infected mice, exposed additionally to filarial infective larvae, cerebral malaria did not develop and their survival was significantly prolonged^70^.

In the group of *D. repens*-infected dogs, expression of *IL-13* was relatively high, and accompanied by low expression of regulatory *IL-10* (and lowest number of alterations in blood parameters). In pulmonary dirofilariasis due to *D. immitis* infection in dogs, the initial inflammatory reaction that occurs in the walls of the pulmonary vasculature is critical in the development of the entire disease process^35^. Similar to *D. repens*, in dogs infected with *D. immitis* both patent infection with circulating MFs and occult infection with no detectable MFs may occur^35^. Dogs with patent *D. immitis* infection had higher expression of circulating *IL-4*, *IL-10* and induced nitric oxide synthase (iNOS) than dogs with occult infection^40^.

Data on the mRNA expression of the different cytokines have shown that both innate and acquired immune responses are present in *D. immitis* infected dogs^35,40^. Important proinflammatory mediators, as iNOs and tumour necrosis factor (*TNF-α*) were consistently present. The expression of Th1-and Th2-related interleukins (*IL-2, IL-4, IL-5*) has also been detected. Regulatory *IL-10* was present in dogs with patent infections, while it was not expressed at detectable levels in amicrofilaraemic dogs. This cytokine has been implicated in the hyporesponsiveness observed in filarioses^35^.

Interestingly, it was demonstrated that *Wolbachia*, bacterial endosymbiont of *Dirofilaria* spp., interacts with *D. immitis*- infected hosts^41^. *Wolbachia* was identified in many organs/cells of *D. immitis*-infected dogs such as renal tubular cells, glomeruli or inflammatory pulmonary cells by the use of immunohistochemistry techniques employing a polyclonal antibody against the *Wolbachia* surface protein (WSP)^41,71^. Moreover, IgG antibodies against WSP have been detected in cats with *D. immitis* infections, in humans diagnosed with pulmonary dirofilariosis, and in healthy, seropositive humans living in areas endemic for heartworm^35^. High titres of specific IgG antibodies have also been detected in dogs with different clinical presentations of pulmonary dirofilariosis^41^. For example, in an amicrofilaraemic dog with massive pulmonary thromboembolism, the IgG response to both *Wolbachia* and *D. immitis* antigens was much higher than in other amicrofilaraemic dogs that were asymptomatic^35^. These findings suggest the extremely complex immune response in dogs infected with *Dirofilaria* spp.

Our study has several limitations. Molecular methods did not allow for detection of the *D. repens* infection in amicrofilaraemic dogs. The study on immune responses is based solely on mRNA detection, and next needs to be further expanded by detection of cytokine/transcription factors and proteins in the sera of dogs with single and double infections. Collection of serum was not possible in the present study (only EDTA-fixed blood samples were available). Furthermore, a selected range of cytokines/transcription factors was used, mainly due to limitation in accessibility of probes and of financial support. Assessment of the degree of expression of other key cytokines/factors such as *IL-2*, *IL-12*, *TNF-α* and *TNF-β* or *SOCS5* could help to more accurately define the immune response profile. For example, in the study by Zygner et al. (2014)^72^ an increase of serum *TNF-α* concentration was observed during canine babesiosis, and this increased *TNF-α* concentration affected the development of hypotension and renal failure in canine babesiosis due to *B. canis*.

## Conclusions

In summary, with just a few mild exceptions, no major alterations were found in blood parameters of dogs infected only with *D. repens*, and these dogs predominantly expressed Th2-related cytokines/factors. Dogs infected with *B. canis* showed mixed expression of immune response cytokines/transcription factors and showed marked pathology. While some features of Th1, T-regulatory and Th2 responses were observed in *B. canis* and *D. repens* co-infected dogs, the Th2-related response appeared to predominate in these animals, and values of biochemical parameters were closer to those of healthy dogs than those solely infected with *B. canis,* suggesting a milder course of babesiosis in these animals. These findings are important due to the current spread of dirofilariosis and babesiosis in central Europe.

## Data Availability

The datasets used and/or analysed during the current study are available from the corresponding author (AB) on reasonable request.
